# Integrated Micro-Devices for a Lab-in-Organoid Technology Platform: Current Status and Future Perspectives

**DOI:** 10.3389/fnins.2022.842265

**Published:** 2022-04-26

**Authors:** Gian Nicola Angotzi, Lidia Giantomasi, Joao F. Ribeiro, Marco Crepaldi, Matteo Vincenzi, Domenico Zito, Luca Berdondini

**Affiliations:** ^1^Microtechnology for Neuroelectronics Laboratory, Fondazione Istituto Italiano di Tecnologia, Genova, Italy; ^2^Electronic Design Laboratory, Fondazione Istituto Italiano di Tecnologia, Genova, Italy; ^3^Department of Electrical and Computer Engineering, Aarhus University, Aarhus, Denmark

**Keywords:** 3D cell cultures technologies, biointerface engineering, biological model, complementary metal-oxide-semiconductor (CMOS), organoids, neural recording, inductive link, radio frequencies (RF) waves

## Abstract

Advancements in stem cell technology together with an improved understanding of *in vitro* organogenesis have enabled new routes that exploit cell-autonomous self-organization responses of adult stem cells (ASCs) and homogenous pluripotent stem cells (PSCs) to grow complex, three-dimensional (3D), mini-organ like structures on demand, the so-called organoids. Conventional optical and electrical neurophysiological techniques to acquire functional data from brain organoids, however, are not adequate for chronic recordings of neural activity from these model systems, and are not ideal approaches for throughput screenings applied to drug discovery. To overcome these issues, new emerging approaches aim at fusing sensing mechanisms and/or actuating artificial devices within organoids. Here we introduce and develop the concept of the *Lab-in-Organoid* (LIO) technology for in-tissue sensing and actuation within 3D cell aggregates. This challenging technology grounds on the self-aggregation of brain cells and on integrated bioelectronic micro-scale devices to provide an advanced tool for generating 3D biological brain models with in-tissue artificial functionalities adapted for routine, label-free functional measurements and for assay’s development. We complete previously reported results on the implementation of the integrated self-standing wireless silicon micro-devices with experiments aiming at investigating the impact on neuronal spheroids of sinusoidal electro-magnetic fields as those required for wireless power and data transmission. Finally, we discuss the technology headway and future perspectives.

## Introduction

Current investigative and therapeutic research in neuroscience largely relies on experimental studies based on biological models that span from *in vitro* cultures of dissociated neurons (Keller and Frega, 2019), *ex vivo* brain slices (Humpel, 2015), and *in vivo* experiments in animal models (Hong and Lieber, 2019) (or any combination thereof). These different models unquestionably permitted to understand some of the fundamental principles of neurobiology and neurophysiology, underlying the complex neuro-dynamics of brain functions in health and disease. However, their intrinsic limitations are also a major source of the challenges faced while progressing in neuroscience and therapeutic research. Planar dissociated neuronal cultures undisputedly enabled a significant advance in our understanding of cell and network behavior, but growing evidence shows that such 2D systems can lead to cell bioactivities that deviate appreciably with respect to *in vivo* response (Abbott, 2003; Weigelt et al., 2014). *Ex vivo* brain slices somehow maintain some of the native network connectivity, but are significantly sensitive to axotomy and neuronal death (Lossi and Merighi, 2018). Further, although undoubtedly offering access to the full system complexity, animal models are inherently characterized by low experimental sample sizes, poor reproducibility and, above all, questionable human translational relevance and sustainability (Rhrissorrakrai et al., 2015). Therefore, there is the need for more human-relevant brain models that can retain a sufficient level of neurobiological complexity and that can facilitate the longitudinal read-out of emerging and externally induced changes in neuro-dynamics and biosignals.

Advancements in stem cell technology together with an improved understanding of *in vitro* organogenesis have enabled new routes for the generation of diverse human cell types and of three-dimensional (3D) organoid models (Rossi et al., 2018). Notably, brain organoids from human pluripotent stem cells (or hPS-derived brain organoids) represent a growing technology to generate a variety of human-derived brain models *in vitro* and have gathered significant attention in the scientific community and public domain over the last years (del Dosso et al., 2020). This is due to their remarkable capability to recapitulate the key steps involved in the neurodevelopment and in the expression of pathophysiological hallmarks, resulting in neural tissues with a surprising degree of similarity to human brain circuits (Wang, 2018) for the study of disease progression (Kyrousi and Cappello, 2020; Shou et al., 2020). They offer unprecedented access to molecular and cellular networks of human neurobiology, with an extraordinary potential for understanding (and in perspective curing) neural development diseases (di Lullo and Kriegstein, 2017), brain cancer (Mariappan et al., 2021), and infectious disorders (Aguilar et al., 2021). Given the exponential development of brain organoids over the recent years, these models are likely to become the mainstream approach to study brain development, function and dysfunction, as well as for addressing the efficacy and safety of potential novel therapeutic treatments for brain disorders.

Despite major advancements in the development and optimization of culture conditions for the generation of different regional brain tissue structures with minimal inter-preparation variability (Velasco et al., 2019), the study of these models is still mainly limited to morphological and gene-expression read-outs. Current neurotechnologies for electrophysiological measures severely limit the capacity to continuously access neuronal signals inside organoids for longitudinal studies, as well as the spatiotemporal resolution of single-cell activity. Briefly, patch-clamp (Li et al., 2017) and optogenetic (Klapper et al., 2017) based techniques enabled the monitoring of single cells at sub-millisecond temporal resolution recordings, while calcium imaging gave access to small cellular aggregations (Sakaguchi et al., 2019) with a coarser spatio-temporal resolution. Finally, planar (Muzzi et al., 2021) or penetrating (Quadrato et al., 2017) multi-electrode arrays (MEAs) have been used respectively for probing organoids superficially or in depth to monitor their electrical activity at specific time-points with sub-millisecond temporal resolutions. Thus, there is a growing need of neurotechnologies for the continuous control and monitoring of the functional development of brain organoids over long periods of time, as required during the maturation phase (up to several months), and for efficiently reading out neural responses to pharmacological, electrical or optogenetic perturbations while implementing functional assays.

Within this framework, an emerging new approach consists in fusing sensing strategies and/or actuating artificial devices with organoids (Li et al., 2019; McDonald et al., 2021). Differently from these approaches, our concept is based on the development of bio-artificial 3D brain model systems with seamless, tissue-integrated, untethered microscale active devices that can provide wireless sensing or actuation of extracellular bio-signals (Angotzi et al., 2018, 2019a; Lecomte et al., 2020). Advances in complementary metal-oxide-semiconductor (CMOS) technology have in fact permitted over the past few years the design and fabrication of low-area, low-power, and highly integrated solutions optimized for untethered sensing of biological parameters. Notably, a number of fully injectable wireless micro-devices have already been successfully validated in animal models for monitoring biological parameters such as temperature, glucose, and even the spiking activity from a mouse brain (see Khalifa et al., 2021), thus supporting the feasibility of the model proposed in this work. Table 1 reports a comparison of the most relevant systems proposed in the literature.

**TABLE 1:** Performance Specifications of our solution in comparison to other State-of-the-Art Systems.

References	Li et al. (2019)	McDonald et al. (2021)	Lee et al., 2018, 2020	Seo et al. (2016)	Khalifa et al. (2019)	This work(*)
Application	Recording	Recording	Recording	Recording	Stimulation	Recording
Communication	Wired to external DAQ	Wired to external DAQ	Wireless optical	Wireless ultrasound	Wireless RF	Wireless RF
Native process	Passive electrodes	Passive electrodes	CMOS 180 nm	N/A	CMOS 130 nm	CMOS 130 nm
Post-processing	N/A	N/A	PVLED on CMOS	Piezo on CMOS	Fully CMOS compatible	Fully CMOS compatible
Power consumption [μW]	N/A	N/A	1	N/A	<50	6.18
Model	*In vitro*	*In vitro*	*In vivo*	*In vivo*	*In vivo*	*In vitro*
	Organoids	Organoids	(mouse CNS)	(mouse PNS)	(mouse CNS)	organoids
Volume [mm^3^]	N/A	N/A	0.0008	2.4	0.009	0.0005

* System not fully released.

**FIGURE 1:**
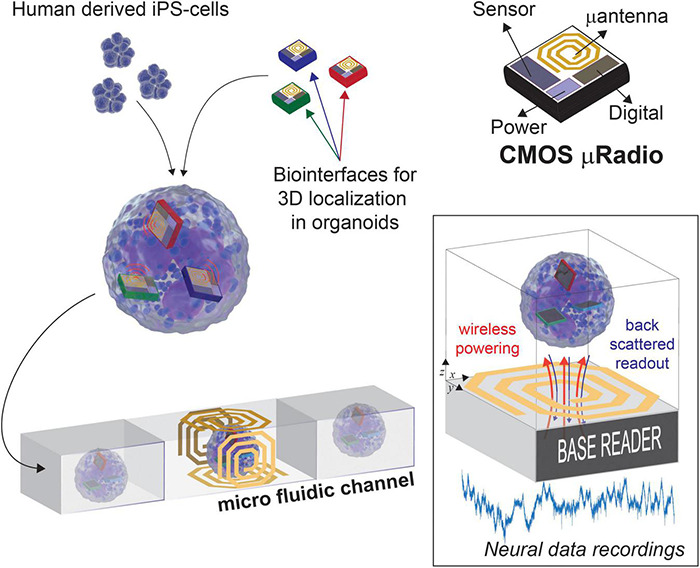
Concept view of *Lab-in Organoids*: active devices seamlessly integrated into developing organoids can be used for monitoring their bioelectrical activity over the entire life span.

As illustrated in Figure 1, we envision the generation of these novel 3D models with built-in transducers by promoting the self-aggregation of cells and microscale devices while culturing *in vitro* the resulting assemblies. In our previous work we have shown that the surface functionalization of Si micro-devices of 100 μm × 100 μm in size can be used to control their final localization inside self-aggregated bio-artificial neural spheroids (Lecomte et al., 2020). For read-out, we plan to channelize each 3D model within a microfluidic device, therefore, with powering and readout operations occurring for one model at a time, while transiting through the section equipped with a *Base reader*. Among the methods available for wireless power and data transmission, ultrasound, optical, and near-field Radio Frequency (RF) are the most promising solutions, as they provide good trade-offs between scalability and power transmission efficiency. The opto-electronic (Lee et al., 2018, 2020) and ultrasound (Seo et al., 2016) transducing modalities, for instance, were demonstrated *in vivo* to be valid methods for delivering enough energy to a neural front-end system capable of capturing and encoding neural data before transmitting it, without causing excessive heating. However, these approaches need *ad hoc* post-processing MEMS fabrication to integrate the required transducers on CMOS. Differently, we target the use of near-field RF field since it can be implemented directly on-chip, thus reducing costs, improving productivity, and providing compact and reliable prototypes (Khalifa et al., 2019). Four orthogonal coils are conveniently placed at the Base reader, on each face of the microfluidic channel, in order to guarantee a minimum distance for the wireless link (few millimeters) and to mitigate possible issues arising from non-perfect alignment with the active devices. Such *lab-in-organoid* (*LIO*) technology would permit to routinely monitor and perturb functional signals inside developing brain organoids for tracking circuit development and levels of maturation, or to deploy organoid based functional assays without cell labeling as needed for microscopy imaging (Rios and Clevers, 2018).

In Section “Materials and methods,” we discuss and evaluate the constraints for the challenging development of such *LIO* technology platform. Through previously reported circuit simulations and experimental studies, we evaluate needs and challenges toward a first CMOS low-power solution that integrates all circuits required for sensing and amplification of bioelectrical signals into a 100 μm × 100 μm die area, while providing RF wireless power delivery and data transmission. In Section “Results,” we provide post-layout circuit simulations on a 130 nm RF-CMOS technology, together with preliminary bench test measurements on a realized prototype and requirements for the RF antennas. The effects of high frequency electromagnetic fields on neural activity and viability have been preliminarily evaluated in neural spheroids to determine the potential operational conditions of such devices. Interestingly, the proposed micro-device platform could be extended to integrate different chemical or physical transducers without requiring modifications on the RF/digital subsystems.

## Materials and Methods

### Complementary Metal-Oxide-Semiconductor Circuit and Principle of Operation

**FIGURE 2:**
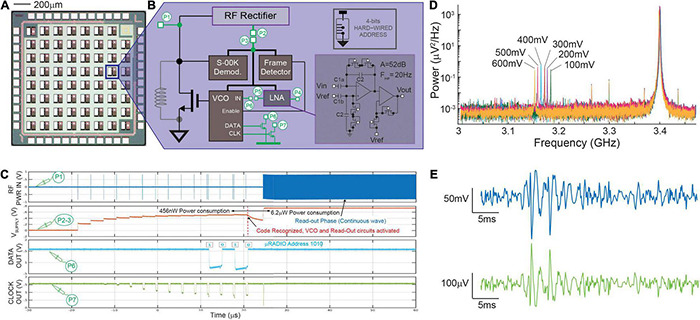
CMOS μRADIO. Panel **(A)** Image of a chip prototype integrating multiple instances of a 100 × 100 μm^2^ μRADIO, two of which include additional circuits for testing purposes. Panel **(B)** block diagram of the μRADIO showing in green test circuits that are connected to IO pads (test point P1–P7) and, inset, circuit schematic of the AC-input low-noise bio amplifier. Panel **(C)** analog waveforms previously reported in Angotzi et al. (2019b) measured on different circuit nodes from a device under test for distinct modes of operation (power up addressing, readout). Panel **(D)** Power distribution of the backscattered signal measured on node P1 for different voltages applied at the input of the voltage controlled oscillator (P6). Panel **(E)** post-layout simulations of the demodulated VCO output (top, blue trace) when the Low Noise Amplifier is stimulated with a synthetic neural trace (bottom, green trace).

With the final goal of achieving distributed bio-sensing from inside the organoids, we have studied the design of a CMOS active μRadio under low-power and low-area constraints. The device is supplied wirelessly through the incident RF signals and communicates data wirelessly through backscattered signals, which can be very efficient for very short-range communications (Han and Wentzloff, 2010). The conceptual block diagram of the proposed micro system is illustrated in Figure 1, while Figures 2A,B show a realized CMOS prototype integrating multiple instances of the μRadio and the circuit architecture of the latter, respectively. Wireless communication and power transfer are achieved by inductor coupling, using a single transistor to clamp the voltage across the receiving inductor for back scattered signal transmission of read-out data. In our implementation, previously introduced in Angotzi et al. (2018), a remote base reader produces an incident sinusoidal wave that can be either continuous (analog) or pulsed (digital). When continuous, the *RF Rectifier* provides a stable power supply to all the internal sub-systems. Conversely, pulsed incident waves can either be used during a bootstrap powering phase to “wake up” the μRadio or to carry digital information such as the physical address of the targeted micro-device which, in current implementation, is hardwired on 4 bits. To decode such digital information, small signal variations of the rectified pulsed signal are amplified and detected using the *Baseband* and *Demodulator* units which implement a Synchronized On-Off Keying (S-OOK) modulation scheme (single and double pulse coding for the logic states zero and one, respectively) with an extremely limited number of logic gates (Crepaldi et al., 2014). When the address corresponding to a specific device is received, it is identified using the *Frame detector* and, as a result, the *Voltage Controlled Oscillator* (*VCO*) is activated, and backscattered communication is enabled. At this stage, the base reader transmits a continuous analog sinusoidal wave with adequate power, and it configures itself in listening mode to detect the impedance variations imposed by the μRadio. To mitigate issues arising from possible non-ideal alignments with the μRadios, we envisage the possibility of equipping the Base reader with multiple orthogonal coils, which can be pooled during the bootstrapping and addressing phases to identify the one that guarantees the best coupling efficiency. With such stable rectification, the *Readout Circuit* generates an analog output voltage that is used to control the VCO and, in turn, to perturb the impedance across the coupling inductor using the dedicated metal-oxide-semiconductor field effect transistor (MOSFET). At the base reader side, the receiver demodulates data and turns off the power when finished. Finally, in the current implementation, the *Readout Circuit* consists of a two-stages low-noise amplifier, whose circuit schematic is shown in the inset of Figure 1. This is suitable for reading out the low amplitude (in the order of μV) bio-electrical potentials of neuronal cells from inside the brain organoids. To remove the large DC offset that might arise at the electrode-tissue interface, the first stage is AC coupled. Similar to the circuit described in Harrison and Charles (2003), the high-pass cut-off frequency is set to 1/(2πR_eq_C_2_) whereas the gain is determined by the capacitance ratio C_1_/C_2_. The second stage consists of a non-inverting amplifier that provides further amplification to secure the adequate signal dynamic range to drive the VCO. The sensing and the reference electrodes are implemented through openings on the insulator over the top plate of the large MIM input capacitors C_1a_ and C_1b_, respectively.

### Electrical Characterization of the μRadio

A CMOS device prototype was fabricated in a standard, commercially available, 130 nm RF-CMOS technology (one poly, eight metal layers, MIM, and MOM caps). The realized chip prototype includes multiple instances of the actual μRadio (see Figure 2A), together with some test circuits, all in a 1.6 × 1.6 mm^2^ silicon area. For these test circuits, access points dedicated to measuring on-chip internal voltages (nodes *P1*–*P7* illustrated in Figure 2B) were routed to the I/O pads. The realized prototypes were wire bonded to full custom printed circuit boards (PCBs) comprising the necessary connectivity for data readout using an oscilloscope (DSO9404A, Agilent, Santa Clara, CA, United States). A microwave RF signal generator (SMB100A, Rohde & Schwarz, Munich, Germany) was used to apply on node *P1* the RF incident sinusoidal waveform with controlled frequency and power, and to synthetize the powering and addressing sequence with S-OOK modulated trains of pulses. Finally, backscattered radiation was detected using a Narda 4202B-10 directional coupler (Narda-MITEQ, Hauppauge, NY, United States): the input port was directly connected to the node *P1*, whereas the coupled and the output ports were connected to the oscilloscope and to a RF signal generator, respectively. All device characterizations reported in section 3.1 were carried out in “wired conditions”, meaning that the RF input was delivered directly to port *P1* of the test μRadio through a SMA connector.

### *In vitro* Culture of Neural Spheroids

All animal procedures carried out in this work were approved by the institutional IIT Ethics Committee and by the Italian Ministry of Health and Animal Care (Authorization No. 110/2014-PR of December 19, 2014).

Primary neuronal spheroids were established from cerebral cortices of Embryonic day 18 (E18) Sprague–Dawley rats and maintained at 37°C in a humidified atmosphere of 5% CO_2_. The following solutions and media were used: Hanks Balanced Salt Solution (HBSS) (Sigma H6648); digestion solution—Trypsin (0.125%, Thermo Fisher Scientific 25050014) in HBSS + DNase (0.25 mg ml^–1^, Sigma D5025) in HBSS 5 mM CaCl_2_; complete Neurobasal medium (NB, Thermo Fisher Scientific 21103049) supplemented with B27 (2%, Thermo Fisher Scientific 17504044), Glutamax (1%, Thermo Fisher Scientific 35050038), and Penicillin/Streptomycin (1%, Sigma P4333); FBS (Sigma F7524). Briefly, embryos were removed and decapitated, brains were extracted from the skulls and placed in cold HBSS. After dissection, cortices were placed in the digestion solution and incubated in water bath at 37°C for 30 min. Few milliliters of complete NB + FBS (10%) were added to the cell solution, centrifuged at 1200 rpm for 5 min, and the supernatant was removed. The cell pellet was suspended in fresh complete NB + FBS (10%) and gently pipetted for no more than 10 times with P1000 pipette. The solution was filtered with a cell strainer (Biologix 15-1040, 40 μm pore size), centrifuged at 700 rpm for 7 min, and the supernatant was removed. The cell pellet was resuspended in complete NB. Cell viability at the time of isolation was determined by a Trypan Blue Exclusion Assay (Sigma T8154). Cortical cells were then seeded at a density of 6,500 cells in 75 μL medium in ultra-low attachment plates (GravityTRAP ULA plate 96-wells). In order to avoid bubbles in the wells, first 25 μL of warm NB was plated in the wells, then the plate was centrifuged at 250 g for 2 min, before adding 6,500 cells in 50 μL in each well. After a few hours, the plate was centrifuged at 250 *g* for 2 min before being kept in incubator for 21 days. At day *in vitro* (DIV) 5, 10, 14, and 19, the medium was partially replaced with 30 μL of fresh complete NB.

### Exposure of Spheroids to Electromagnetic Fields

To evaluate the impact of sinusoidal RF electromagnetic fields on neuronal spheroids, we conceived and realized a custom experimental setup. A petri dish containing the spheroids and filled with cell culture medium was placed between two antennas connected to Port 1 and Port 2 of a vector network analyzer (VNA - E5071C, Agilent, Santa Clara, CA, United States). In order to ensure field uniformity, the two antennas were designed as circular copper plates with a radius (25 mm, resulting in an EM field of about 500 μW/cm^2^) much larger than the size of the petri dish (10 mm radius) and of the separation between the antennas (5 mm). Finally, the temperature of the culture medium was monitored during the exposure to the EM filed using a PT100 temperature sensor (SFIL1, Hanna Instruments). The VNA was set to produce a 10 dBm output power on Port 1 with a continuous frequency sweep in the range from 2 to 6 GHz. The power of the EM field between the two antennas was derived from the S_21_ scattering parameter (S-parameters), whereas the total energy was derived from the signal power by considering the exposure time.

For these experiments 6 groups of samples were considered, each containing 8 spheroids. A total of 3 groups were exposed to the EM field for increasingly longer periods of time, of either 15, 30, or 60 min. The remaining 3 groups were used as control groups (one was always kept in the incubator, while the other two were kept outside of the incubator as for the samples treated with EM fields for the longer exposure times of 30 and 60 min).

### Immunofluorescence Imaging and Analysis

After exposure to the EM field, spheroids (DIV 21) were fixed in paraformaldehyde (PFA, 4% v/v, Santa Cruz Biotechnology 30525-89-4) for 2 h. All the following steps were performed on a shaker at 4°C. The following antibodies were used: rabbit anti-cFos (Cell Signaling 2250, 1:500) and Alexa488 goat anti-rabbit (Invitrogen A11034, 1:1000). Spheroids were permeabilized and blocked with Triton X-100 (TX, 0.2%, Sigma T9284) and bovine serum albumin (BSA, 10%, Sigma A9647) in PBS for 1 h, and subsequently incubated in primary antibodies diluted in 0.2% TX and 3% BSA in PBS (B-PBT) overnight. Spheroids underwent 3 washes (15 min each) with 0.2% TX in PBS (PBT) before incubation with secondary antibodies in B-PBT for 2 h. After that, spheroids underwent 3 washes (15 min each) with PBT and then were incubated with Hoechst (BD Biosciences 561908, 1:300) in PBT for 1 h and returned to PBS where they were kept before being transferred to glass-bottomed confocal dishes for imaging. All images were acquired with 40x objective lenses using a Leica SP5 inverted confocal microscope (Leica Microsystems). For each condition, the maximum intensity projection of a Z-stack from approximately half spheroid (from the bottom to the maximum diameter) is displayed. The quantification was done by counting c-Fos^+^ cells and nuclei from each confocal image using ImageJ software.

### MTT Assay

After the exposure to the EM field, spheroids dedicated to MTT assays were placed in a 96-wells plate (1 spheroid/well) and in a solution containing NB + 3-(4,5-dimethylthiazol-2-yl)-2,5-diphenol tetrazolium bromide (MTT; Sigma M2128) was added. MTT was previously diluted 5 mg ml^–1^ in PBS. The plate was kept 3 h in incubator. Then, the solution was removed, and the formed formazan crystals were dissolved in 2-propanol. Absorbance was measured at 570 nm, using 650 nm as the reference wavelength (Tecan, Männedorf, Switzerland). The percentage of cell viability was assessed as Abs_T_/Abs_C_ × 100, where Abs_T_ and Abs_C_ are the absorbance of “treated” and “control” spheroids, respectively.

### Statistical Analysis

For the immunofluorescence analysis and MTT assay, statistical analysis of the data was performed using Prism 5 (GraphPad, San Jose, CA, United States) for the number of spheroids for each experiment indicated in the figure legends. Means and standard deviations were reported for all experiments. ANOVA followed by Bonferroni *post hoc* comparison was used. Values were considered significantly different at *P* < 0.05.

## Results

### Complementary Metal-Oxide-Semiconductor μRadio Simulations and Bench Test Measurements

As mentioned in Section “Electrical Characterization of the μRadio,” the circuits were designed in a 130 nm CMOS-RF technology process under stringent low-power and low-area constraints. Computer aided design (CAD) pre- and post-layout simulations were performed and confirmed the feasibility to integrate all the circuits for RF powering, data transmission, and bioelectrical signal readout on a total device area of 100 μm × 100 μm with a power consumption of 6.18 μW in read-out mode. More in detail, the *Baseband* unit, the *Readout Circuit* and the *Frame detector* occupy the largest areas (30, 35, and 18% of the total area, respectively), whereas the *VCO* and the *S-OOK demodulator* require a smaller area (0.025% and 10%, respectively). For the layout, the two top metal layers available from the RF-CMOS process design kit were used to implement the MIM capacitors (C1 and C2) required by the low-noise amplifier, the next three metal layers (M6 to M4) were used to implement an integrated spiral inductor, whereas the remaining three metal layers were used for routing. The power consumption of the μRadio during the three consecutive working phases (bootstrap powering, addressing and read-out) amounts to 270 nW, 456 nW, and 6.18 μW, respectively. These results have been also confirmed by bench electrical measurements on the prototype.

Figure 2B shows the block diagram of the μRadio, with nodes *P1*–*P7* (setup described in Section “Electrical Characterization of the μRadio”) routed to IO bonding PADS, in order to perform the device electrical characterization, as previously described in Angotzi et al. (2019b). As reported in Figure 2C, during the bootstrap powering phase, 10 trains of RF pulses with a center frequency of 3.4 GHz, duration of 200 ns, and a peak power of 18 dBm were delivered to port *P1* through a SMA connector, causing the increase of the output voltage of the RF rectifier (node *P2* shorted off-chip to node *P3*) up to about 600 mV. During the subsequent addressing phase, the physical address of the target μRadio (‘1010’ in the example reported here) was produced and correctly recognized by the S-OOK demodulator (node *C*). Matching with the hardwired address by the *Frame detector* enabled the *VCO* and the *Readout Circuit*, with a consequent sudden voltage drop of the power supply that was rapidly recovered during the subsequent readout phase, that is, when a continuous RF signal (still with a 3.4 GHz center frequency) permitted the generation of an on-chip power supply of about 800 mV. Furthermore, the operation of the on-chip VCO was validated by showing that, when stimulated with different DC voltage levels in the 100–600 mV range (signal applied to port *P6*), the backscattered signal produced a proportional frequency shift of about 15 MHz per 100 mV of the input signal amplitude (see Figure 2D). Because of the low-power and low-area design constraints, the low noise amplifier was optimized to drive the VCO input and not the IO bonding pad on port *P5*. Therefore, the electrical characterization of the *Readout Circuit* alone was not possible. For this reason, we report here CAD post-layout simulations instead where a synthetic neural trace amplified by the *Readout Circuit*, is modulated in frequency by the *VCO* (Figure 2E). In our first prototype, capacitors C_1_ and C_2_ where respectively sized as 45 × 90 μm^2^ (4 pF) and 4 × 20 μm^2^ (85 fF) for a total gain of the first stage amplifier of about 33 dB, and a high pass cut-off frequency of 30 Hz. The second stage amplifier provides 19 dB of additional gain for a total gain of 52 dB. It is worth noting that the relatively low gain and output dynamic range (400 mV) of the second stage amplifier permit to avoid signal distortions from the non-linear behavior of the subthreshold-biased diode-connected transistors that implement the non-inverting stage, which is exacerbated for large voltage swings. Finally, the input referred noise, integrated in the 1–5 kHz frequency band, accounts for 12 μV_RMS_, while the two operational amplifiers occupy a silicon area of 27 × 100 μm^2^ and consume 480 nW of power when supplied at 600 mV.

Finally, it is worth mentioning that the design of the integrated inductors of the power up circuitry of the μRadios within the prototype was not yet optimized. The inductor was designed using three metal layers, with an outer diameter of 75 μm, 5.5 turns, line thickness of 2 μm, and an interspace between line of 280 nm. Based on the technology RF design kit, it results in a capacitive reactance which leads to an inductance of −14 nH. The experimental measurements showed that, with this implementation, the inductive link permitted reaching a maximum voltage on node ***B*** of only 250 mV while an 18 dBm source power delivered to port *P1* through a SMA connector was required to secure the correct voltage level of 600 mV. These results suggest the need of major efforts not only for the optimal design of the integrated coil, but also for the matching network to minimize the ratio of reflected power at port *P1*. These design aspects will be addressed in the next prototype implementation to optimize the power transmission efficiency (PTE).

### Biological Effects of Electromagnetic Fields on Neuronal Spheroids

An important aspect that needs to be considered for the development of wireless micro-devices is the possible biological effect on neuronal systems of the RF EM field. To evaluate this, spheroids have been exposed to the influence of the EM field for different periods of time (15, 30, and 60 min) while monitoring the temperature of the culture medium without observing appreciable changes. Induced effects on neural activity and cellular viability were evaluated in subsequent analysis. Controls (i.e., not exposed to the EM field) included a group of spheroids that was always kept inside the incubator (C1), as well as groups that were kept outside the incubator for 30 (C2) and 60 (C3) min, as for spheroids subjected to EM fields for the longer exposure duration.

**FIGURE 3:**
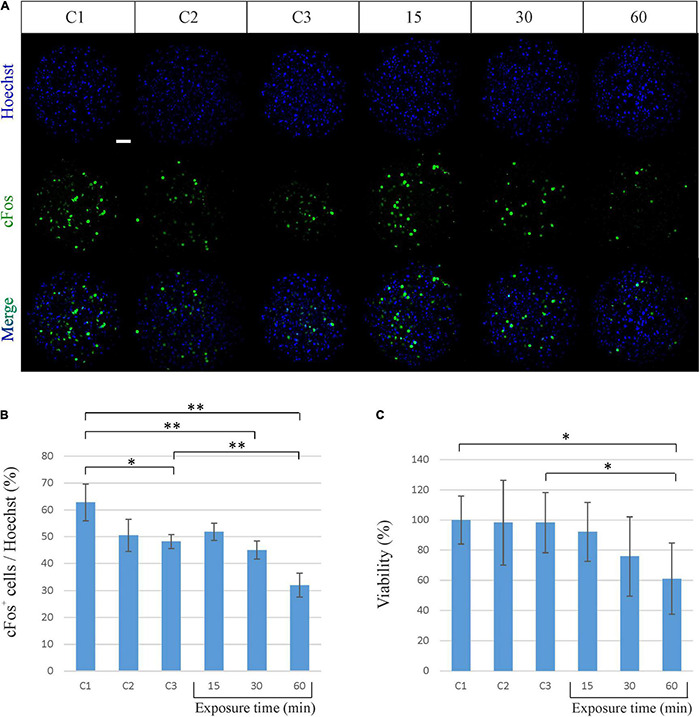
Biological effects of sinusoidal RF EM field (2–6 GHz) on neuronal spheroids activity and viability. **(A)** Representative confocal immunofluorescence images of neuronal spheroids used as controls (C1, C2, C3) and spheroids exposed to the EM field for 15, 30, and 60 min. Spheroids were stained against cFos (green) and all cell nuclei (Heachst, blue). Scale bar: 50 μm. **(B)** Quantitative analysis showing the percentage of cFos-positive (cFos^+^) cells out of the total cells (Heachst). The graph shows the mean ± SD, *n* = 4–6 per group. *P < 0.05; ^**^P < 0.01. **(C)** Percentage of viable neuronal cells after exposure to the EM field for 15, 30, and 60 min. The graph shows the mean ± SD of three experiments performed separately with eight spheroids per group per experiment. *P < 0.05. C1, control spheroids, always kept in the incubator; C2, control spheroids, kept outside the incubator for 30 min; C3, control spheroids, kept outside the incubator for 60 min.

In order to investigate whether the exposure to the EM field sweeping in the frequency range from 2 to 6 GHz might affect the spheroid neuronal activity, we first performed an immunofluorescence analysis of the expression of the cFos neuronal activity early gene. The expression of cFos in neurons is a useful and popular marker of activated neurons. Its basal expression is low in most neurons, but it can be rapidly induced by a broad range of stimuli. The most common approach is to visualize the presence of cFos within nuclei of neurons using immunofluorescence (Krukoff, 1999). As shown in Figures 3A,B, cFos expression does not change after 15 min of exposure to the EM field, while decreases after 30 min of exposure and becomes even lower after 60 min (45% of cFos^+^ cells after 30 min and 32% after 60 min of exposure). It has to be noted that the decrease of cFos expression after 60 min of exposure is also significant compared to its own control C3, in which spheroids were kept outside of the incubator for the same period of time. This indicates that the exposure time to the RF EM field modulates cFos expression.

We then evaluated the effect produced by the EM field on cell viability by performing a MTT assay. Such colorimetric assay is largely used to measure cellular metabolic activity as in indicator of cell viability, proliferation and cytotoxicity. It is based on the capability of metabolically active cells to reduce a yellow tetrazolium salt (MTT) to purple formazan crystals (Mosmann, 1983). As shown in Figure 3C, the exposure of spheroids to the EM field induces a decrease in cell viability that becomes significant after 60 min (decrease of 38.88% for the exposure time of 60 min). It has to be noted that the decrease in cell viability after 60 min of exposure is also significant compared to its own control C3. This underlines that the cell death is effectively due to the exposure to the field and suggests that the constant sinusoidal EM field can have a significant biological effect on cortical neuronal spheroids in a time-dependent (i.e., power-dependent) manner.

## Discussion

Within less than a decade, brain organoids technology has gathered the interest of the Neuroscience community, due to its high potential to improve significantly our understanding of the development and disorders of the human brain. Along with studies on new *in vitro* protocols for inducing human pluripotent stem cells (hPSCs) to reproducibly differentiate into various type of human neural cells (Oh and Jang, 2019), most traditional electrophysiology techniques have been applied to brain organoids so far.

However, conventional optical and electrical neurophysiological techniques applied to acquire functional data from brain organoids are not adequate to chronically record neural activity from these model systems, and are not ideal approaches for high throughput screenings, the hallmark in drug discovery and biology. Interestingly, the adoption of flexible electronics has been recently proposed to achieve seamless neural interfacing and chronic recording from organoids (see Hong et al., 2018; Li et al., 2019; McDonald et al., 2021). Although the extremely small footprint and the minimal electrode-tissue mechanical mismatch are indeed both key factors to minimize inflammation and improve long-term biocompatibility, these solutions still require hardwire connections of each single organoid to a front-end recording system, which might limit its exploitation for screenings.

Along with this concept of generating bio-artificial hybrids to achieve chronic in-tissue electrophysiological recordings, we envision the use of untethered micro-devices to realize *lab-in-organoid* platforms that could solve critical issues related to the integration, powering-up and communication of networks of sensors/actuators placed inside the organoids. Our preliminary results discussed in this work demonstrate that, to reach this goal, cross-disciplinary studies are required, in the fields of electronics, (bio) material science, and Micro Electro-Mechanical Systems (MEMS) manufacturing.

### Complementary Metal-Oxide-Semiconductor Electronics for Micro-Scale Devices

To allow 3D cell growth and formation of spheroids/organoids around artificial devices, the volume for the micro-device must be minimized while power consumption must be sufficiently low to limit local heating that can potentially alter the physiology of the organoid or ultimately lead to cell death. Considering that brain organoids can be as large as few millimeters in radius, we targeted a device volume of 100 × 100 × 50 μm^3^. Also, circuits were mostly biased in weak inversion with a power supply limited to 0.6 V. Wireless powering via a resonance-based inductive link (Khalifa et al., 2019) was preferred to other approaches, such as optical (Chowdhury et al., 2018; Lee et al., 2018, 2020) and ultrasound solutions (Basaeri et al., 2016) for its power delivery capability, reliability and, notably, for its monolithic integration together with other electronic components (Lee and Ghovanloo, 2019). Furthermore, the inductive link can also be exploited for backscattered signal communication [as demonstrated in Kiourti et al. (2016) with solution requiring a footprint in the square centimeters range] in which the information is coded by load modulation without requiring additional RF signal generation inside the micro-device, which would require larger power and further design complexity, exacerbating dramatically the challenges on feasibility and leading to micro-devices with larger die size.

As anticipated in Section “Complementary Metal-Oxide-Semiconductor μRadio Simulations and Bench Test Measurements,” the design of the integrated inductor requires further study and optimized design. In our current implementation, the design of the coils was not finalized since we first aimed at evaluating how sinusoidal EM field would affect the neuronal spheroids. Nevertheless, the present version of the CMOS design discussed in this work permitted to assess the feasibility of integrating into a small silicon area all the modules required by our architecture, while also meeting low power and low noise operation. In particular our results confirmed that (i) a pulsed, incident sine wave can be used for bootstrap powering at start up and for addressing a specific μRadio; (ii) the RF rectifier is capable of producing a stable power supply up to 800 mV when a continuous incident sine wave is directly injected on port *P1*, within a quite large range of center frequencies (1–4 GHz in simulations); (iii) the voltage controlled oscillator can be used to clamp the voltage across the receiving inductor (port P1) thus enabling back scattered signal transmission of data amplified by the Readout circuitry. To identify the most suitable power level of the RF signals capable to secure wireless powering and readout operations, in our next studies we will carry out experimental investigations on integrated antennas with different effective areas. Considering that the power attenuates by the inverse 6th in power and assuming an ideal case of operation in empty space, perfect antenna impedance matching and 100% PTE, a minimum source power of about 60 μW (−12 dBm) will be required from the base reader to operate the μRadio in read-out mode (when it consumes less than 10 μW of power). This requirement is indeed well below the 10 dBm of power used for the incident sinusoidal EM field that we used in our experiments with spheroids, leaving enough room to compensate a power transmission efficiency (PTE) as low as 1%. Anyways, despite on-chip antennas on silicon are prone to a lower efficiency compared to those on high-resistivity substrates, the close proximity between the base reader and each spheroid at one time is expected to alleviate the low efficiency issue. Indeed, as illustrated in Figure 1, each spheroid will be channelized within a microfluidic pipeline and powering and readout operations will occur for one spheroid at a time, while transiting through the section equipped with the reader. Thereby, considering a power level suitable to secure communication, the RF signals are localized in space to the region of the same spheroid. One after the other, the spheroids will flow through the microfluidic pipeline on a time division sequencing, according to the monitoring needs and desired readout strategy. Therefore, the exposure time of one spheroid will not cumulate to the exposure time of the other spheroids. Also, the time division sequencing will not limit the number of spheroids that can be populated within the overall system, assuming that there will be enough microfluidic channels equipped with readers such the desired monitoring strategy can be implemented in due time.

The post-processing of the CMOS micro-device, recurring to MEMS manufacturing technologies, besides the final micro-device dimensions, can also allow for the encapsulation of the micro-device with biologically compatible and functionalized materials, as well as for depositing noble, bio compatible materials on the native Al-Cu CMOS alloy of the sensing electrodes. Solutions previously developed in our lab (see Angotzi et al., 2019a; Lecomte et al., 2020; Ribeiro et al., 2021) for post-processing CMOS dies and (bio)materials functionalization can be adapted for extracting single μRadios from the bulky silicon either from single dies or from whole CMOS wafers. Furthermore, using author’s previous experience with polyimide based neural interfaces (Simi et al., 2014; Rodrigues et al., 2020; Pimenta et al., 2021), polyimide-based 3D structures with through polymer vias (Hussain and Hussain, 2016) can be used to integrate the designed antenna between polyimide layers, routing the electrodes to the top surface of the micro-device ensuring the biocompatibility.

### Radio Frequency EM Field Effects on Spheroid

Over the past few decades, considerable evidence has shown that EM fields can induce biological changes both *in vivo* and *in vitro*, including gene expression, cell proliferation, and nerve cell function, although the mechanisms responsible for such effects are not fully understood and have been the subject of debate (D’Agostino et al., 2018). It is thought that the effects of EM fields are diverse and dependent on the strength, frequency, and duration of the EMF exposures (Banik et al., 2003).

Different studies focused on the effect produced by EM field of extremely low frequencies in the range of 1–300 Hz (Saito et al., 2018; López et al., 2019). Currently, due to the development of new technologies (such as the high-band 5G technology) and novel forms of communication, the concerns about the effect produced by exposure to EM fields also includes extremely high frequencies, in the range of 40–130 GHz (Le Quément et al., 2012; Romanenko et al., 2014; D’Agostino et al., 2018).

In our study, we focused on the biological effects of EM fields with a frequency in the range of 2–6 GHz, as RF carriers in this range may lead to effective tradeoffs between low power consumption, growing with frequency, and compact antenna and then chip size, reducing as the frequency arises. The results reported in this work suggest the existence of reliable time windows within which the biological activity of the spheroids and their cellular viability is very likely not influenced by the exposure to GHz sinusoidal signals, which can be used as carrier signals for powering wirelessly the micro-devices inside the spheroids, and for reading out wirelessly the data related to their biological activity by means of backscattered signal modulation. In detail, for an exposure time of 15 min on spheroids of an age of 21 DIVs, the biological activity and cellular viability are not significantly influenced by exposure to the sinusoidal EM fields. Differently, the variation of the biological activity and the cellular viability with respect to the control group becomes statistically significant for exposure times exceeding 30 min.

The continuous-time exposure adopted in our experiments must be considered as a kind of “stress test,” since powering and readout operations in real conditions will occur with a very low duty cycle with respect to the typically long time constants involved in the time evolution of the biological activity of the spheroids (weeks or even months). Also, it is worth emphasizing that the power level of +10 dBm (i.e., 10 mW, corresponding to about 707 mV on a 50 Ω source resistance) used in our stress-tests is a quite robust signal compared to the signals with a lower power expected for operations in the real scenario with a very short communication range a few millimeters, given the close proximity to the spheroid guaranteed by a proper design of the microfluidic channel. Despite this value may appear as quite high at the current stage of the overall system development, the actual power level in the real scenario will depend on the effective area of the on-chip antenna of the micro-device, as discussed in Section “Complementary Metal-Oxide-Semiconductor Electronics for Micro-Scale Devices.”

## Conclusion

Organoids offer one of the most promising platforms for studying the human brain *in vitro*. Production, studies and exploitation of such brain organoids can benefit from accommodating tools that allow real-time monitoring of signals indicating physiological conditions such as pH, glucose levels, temperature, cell-level signals and, bioelectrical activity. Key aspects toward the concept of lab-in-organoids are the possibility of (*i*) realizing low-power active, untethered micro-devices of small volume, (*ii*) promoting the self-integration of such micro-devices inside forming 3D organoids, and, (*iii*) controlling the final 3D localization of one or more micro-devices inside fully developed organoids to permit multipoint bio-sensing. With respect to these points, the state of our development reported in this work confirms the feasibility of integrating into small silicon volumes all the circuits required for RF power delivery and bidirectional data transmission. Additionally, Micro Electro-Mechanical Systems (MEMS) solutions previously developed in our lab (Angotzi et al., 2019a) for post-processing CMOS dies can be adapted to extracting single μRadios from the bulky silicon either from single dies or from whole CMOS wafers. Similarly, our results previously reported in Lecomte et al. (2020) demonstrate how the presence of Si micro-device of similar size as the one targeted in this work does not affect the developing 3D morphology, cellular composition and the spontaneous neural activity of developing neurospheroids. In addition, surface functionalization *via* protein-binding can tune the integration and final 3D location of self-standing micro-devices into neurospheroids.

In this preliminary study, we have carried out experiments with the objective to identify the viability of the system concept, prior to proceeding with the design and implementation as an integrated microsystem. This experimental study provided important results in the cell viability and preliminary design considerations. Beyond the current developments and the scope of this manuscript, further aspects at different levels, from the design level to the monitoring strategy, should be also investigated, and we will address in our next works. Among these, one major aspect relates to the change of biological activity of neuronal spheroids reported for exposures to sinusoidal EM longer than 30 min. This suggest the need of additional studies to assess if and to which extent such 3D biological models are affected by EM fields. However, it should be mentioned that the resilience of the biological activity and cellular viability of the spheroids to the exposure of the RF signals might depend on the age of the spheroids, as those in advanced development stages could be likely more resilient than those in early-stage developments. This means that the readout strategy can take into account also the development age, and spheroids in a more developed stage could go be readout more frequently, with no or low side effects on their biological activity and cellular viability. Additionally, experimental investigations with a discrete set of single-tone RF signals closely spaced (e.g., 100–250 MHz) shall be conducted in order to characterize the biological activity and cellular viability versus frequency over the entire range 2–6 GHz, for different exposure times at different DIVs. The measured S-Parameters will also allow profiling the power absorption peaks of the spheroids vs. frequency. This information will allow identifying the frequency tones that may lead to reduced power absorption by the spheroids, which means reduced interaction (i.e., further “decoupling”) between the RF signals and the biological activity and cellular viability. The results emerging from this study will allow selecting the most effective operating frequency of the carrier signals for further extending the exposure times with no or low side effects on the biological activity of the spheroids and drive the design and optimization of the on-chip antenna. Finally, studies on the penetration depth of the radiofrequency signals within the spheroids are required in order to evaluate the shielding effect toward the on-chip antenna and the effect of the cell culture medium within the microfluidic channel.

## Data Availability Statement

The raw data supporting the conclusions of this article will be made available by the authors, without undue reservation.

## Ethics Statement

The animal study was reviewed and approved by IIT Ethics Committee and by the Italian Ministry of Health and Animal Care (Authorization No. 110/2014-PR of December 19, 2014).

## Author Contributions

GA wrote the manuscript with support from LG and DZ and inputs from all authors. LG and JR carried out experiments on spheroids. MV performed data analysis. MC and GA designed the CMOS prototype and performed the electrical measurements. GA, MC, and DZ designed the experimental test setups. LB conceived the *LIO* technology and supervised the project.

## Conflict of Interest

The authors declare that the research was conducted in the absence of any commercial or financial relationships that could be construed as a potential conflict of interest. The handling editor declared a past co-authorship with one of the authors MC.

## Publisher’s Note

All claims expressed in this article are solely those of the authors and do not necessarily represent those of their affiliated organizations, or those of the publisher, the editors and the reviewers. Any product that may be evaluated in this article, or claim that may be made by its manufacturer, is not guaranteed or endorsed by the publisher.
